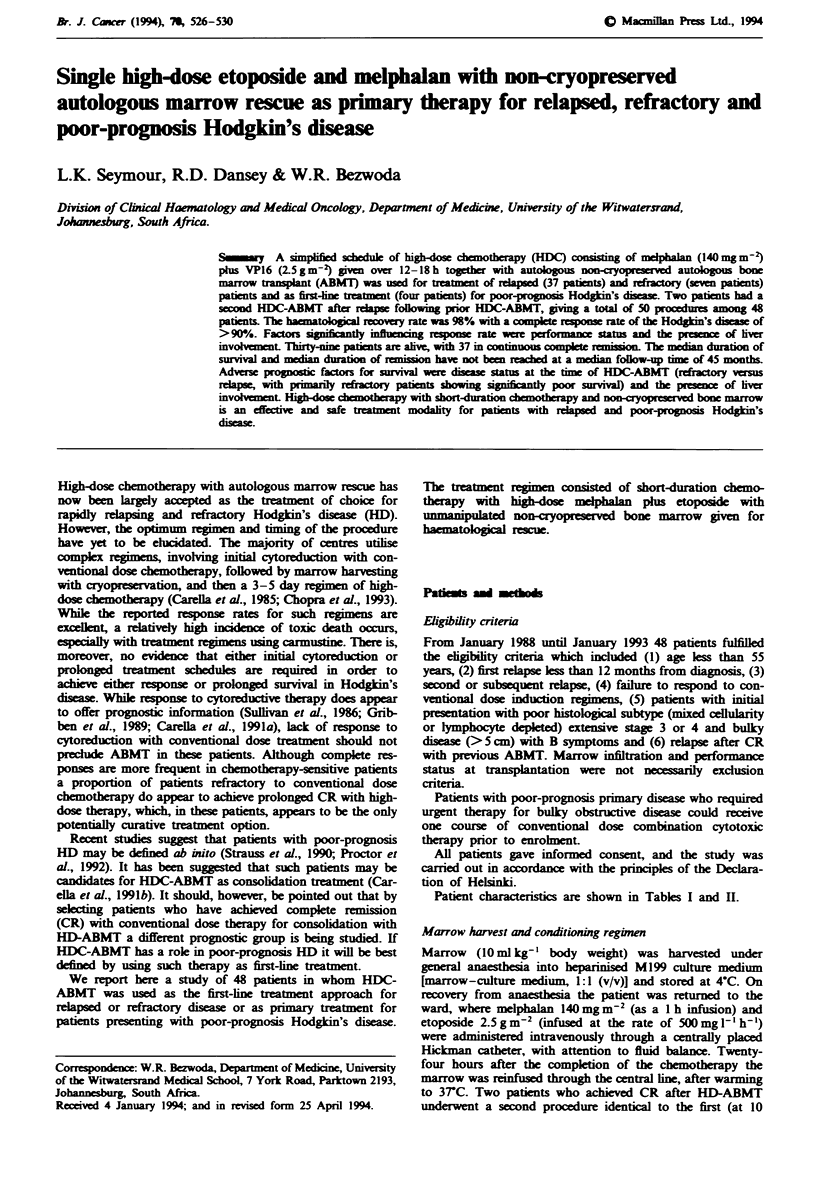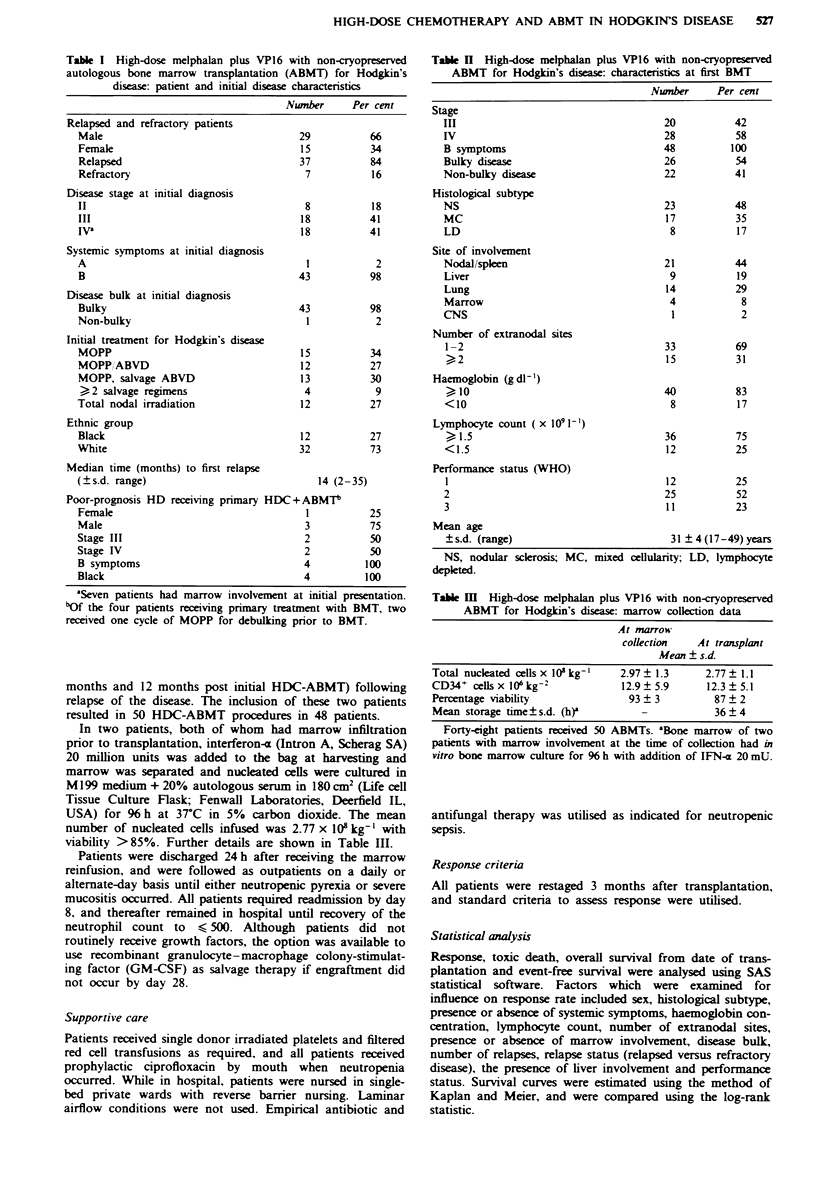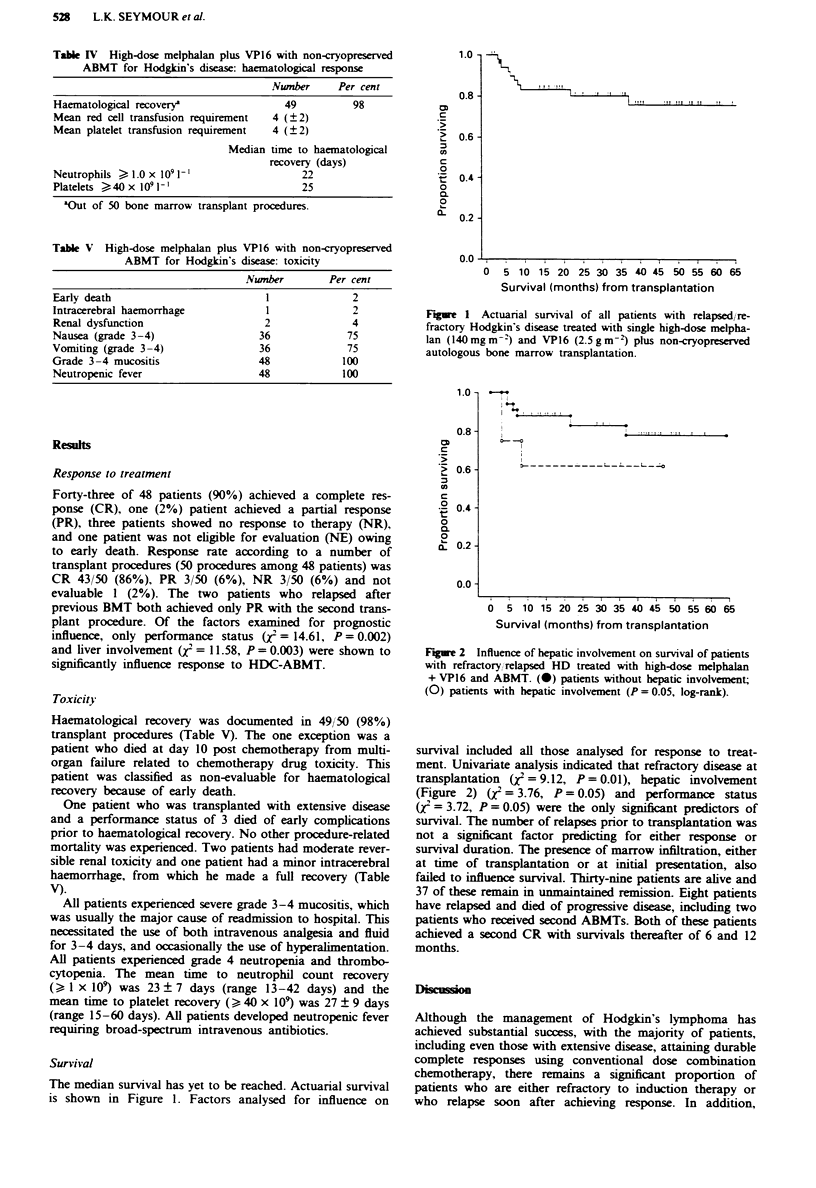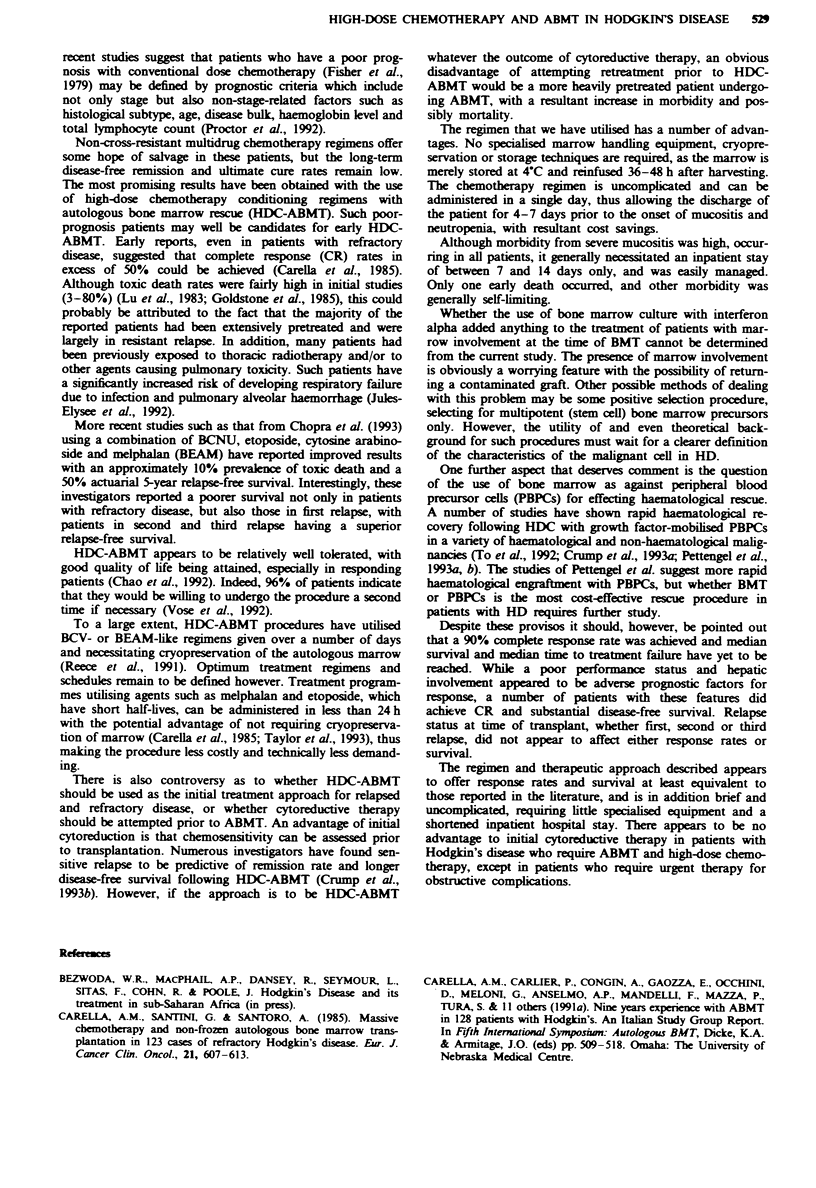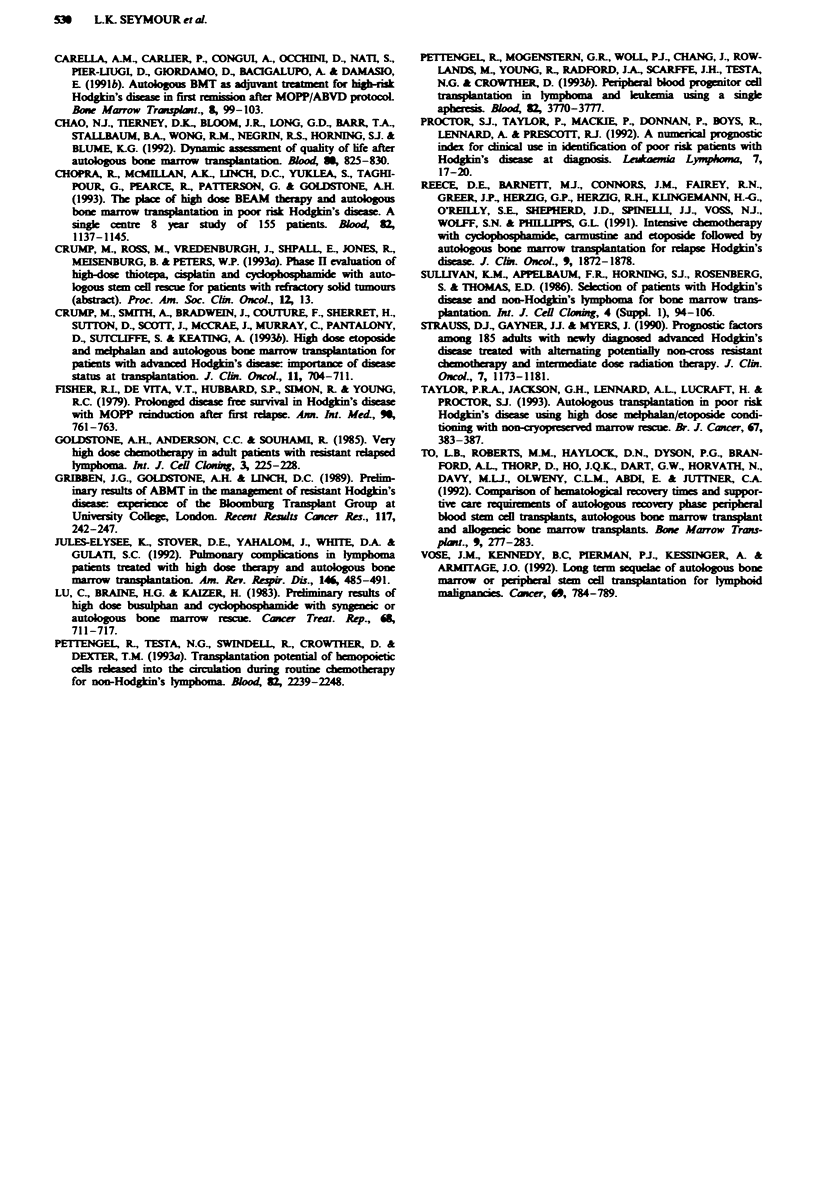# Single high-dose etoposide and melphalan with non-cryopreserved autologous marrow rescue as primary therapy for relapsed, refractory and poor-prognosis Hodgkin's disease.

**DOI:** 10.1038/bjc.1994.339

**Published:** 1994-09

**Authors:** L. K. Seymour, R. D. Dansey, W. R. Bezwoda

**Affiliations:** Department of Medicine, University of the Witwatersrand, Johannesburg, South Africa.

## Abstract

A simplified schedule of high-dose chemotherapy (HDC) consisting of melphalan (140 mg m-2) plus VP16 (2.5 g m-2) given over 12-18 h together with autologous non-cryopreserved autologous bone marrow transplant (ABMT) was used for treatment of relapsed (37 patients) and refractory (seven patients) patients and as first-line treatment (four patients) for poor-prognosis Hodgkin's disease. Two patients had a second HDC-ABMT after relapse following prior HDC-ABMT, giving a total of 50 procedures among 48 patients. The haematological recovery rate was 98% with a complete response rate of the Hodgkin's disease of > 90%. Factors significantly influencing response rate were performance status and the presence of liver involvement. Thirty-nine patients are alive, with 37 in continuous complete remission. The median duration of survival and median duration of remission have not been reached at a median follow-up time of 45 months. Adverse prognostic factors for survival were disease status at the time of HDC-ABMT (refractory versus relapse, with primarily refractory patients showing significantly poor survival) and the presence of liver involvement. High-dose chemotherapy with short-duration chemotherapy and non-cryopreserved bone marrow is an effective and safe treatment modality for patients with relapsed and poor-prognosis Hodgkin's disease.


### Single high-dose etoposide and melphalan with non-cryopreserved autologous marrow rescue as primary therapy for relapsed, refractory and poor-prognosis Hodgkin's disease

L.K. Seymour, R.D. Dansey & W.R. Bezwoda

*Division of Clinical Haematology and Medical Oncology, Department of Medicine, University of the Witwatersrand, Johannesburg, South Africa.*

**Summary** A simplified schedule of high-dose chemotherapy (HDC) consisting of melphalan (140 mg m$^{-2}$) plus VP16 (2.5 g m$^{-2}$) given over 12-18 h together with autologous non-cryopreserved autologous bone marrow transplant (ABMT) was used for treatment of relapsed (37 patients) and refractory (seven patients) patients and as first-line treatment (four patients) for poor-prognosis Hodgkin's disease. Two patients had a second HDC-ABMT after relapse following prior HDC-ABMT, giving a total of 50 procedures among 48 patients. The haematological recovery rate was 98% with a complete response rate of the Hodgkin's disease of >90%. Factors significantly influencing response rate were performance status and the presence of liver involvement. Thirty-nine patients are alive, with 37 in continuous complete remission. The median duration of survival and median duration of remission have not been reached at a median follow-up time of 45 months. Adverse prognostic factors for survival were disease status at the time of HDC-ABMT (refractory versus relapse, with primarily refractory patients showing significantly poor survival) and the presence of liver involvement. High-dose chemotherapy with short-duration chemotherapy and non-cryopreserved bone marrow is an effective and safe treatment modality for patients with relapsed and poor-prognosis Hodgkin's disease.

High-dose chemotherapy with autologous marrow rescue has now been largely accepted as the treatment of choice for rapidly relapsing and refractory Hodgkin's disease (HD). However, the optimum regimen and timing of the procedure have yet to be elucidated. The majority of centres utilise complex regimens, involving initial cytoreduction with conventional dose chemotherapy, followed by marrow harvesting with cryopreservation, and then a 3-5 day regimen of high-dose chemotherapy (Carella *et al.*, 1985; Chopra *et al.*, 1993). While the reported response rates for such regimens are excellent, a relatively high incidence of toxic death occurs, especially with treatment regimens using carmustine. There is, moreover, no evidence that either initial cytoreduction or prolonged treatment schedules are required in order to achieve either response or prolonged survival in Hodgkin's disease. While response to cytoreductive therapy does appear to offer prognostic information (Sullivan *et al.*, 1986; Gribben *et al.*, 1989; Carella *et al.*, 1991*a*), lack of response to cytoreduction with conventional dose treatment should not preclude ABMT in these patients. Although complete responses are more frequent in chemotherapy-sensitive patients a proportion of patients refractory to conventional dose chemotherapy do appear to achieve prolonged CR with high-dose therapy, which, in these patients, appears to be the only potentially curative treatment option.

Recent studies suggest that patients with poor-prognosis HD may be defined *ab inito* (Strauss *et al.*, 1990; Proctor *et al.*, 1992). It has been suggested that such patients may be candidates for HDC-ABMT as consolidation treatment (Carella *et al.*, 1991*b*). It should, however, be pointed out that by selecting patients who have achieved complete remission (CR) with conventional dose therapy for consolidation with HD-ABMT a different prognostic group is being studied. If HDC-ABMT has a role in poor-prognosis HD it will be best defined by using such therapy as first-line treatment.

We report here a study of 48 patients in whom HDC-ABMT was used as the first-line treatment approach for relapsed or refractory disease or as primary treatment for patients presenting with poor-prognosis Hodgkin's disease. The treatment regimen consisted of short-duration chemotherapy with high-dose melphalan plus etoposide with unmanipulated non-cryopreserved bone marrow given for haematological rescue.

Correspondence: W.R. Bezwoda, Department of Medicine, University of the Witwatersrand Medical School, 7 York Road, Parktown 2193, Johannesburg, South Africa.
Received 4 January 1994; and in revised form 25 April 1994.

### Patients and methods

### Eligibility criteria

From January 1988 until January 1993 48 patients fulfilled the eligibility criteria which included (1) age less than 55 years, (2) first relapse less than 12 months from diagnosis, (3) second or subsequent relapse, (4) failure to respond to conventional dose induction regimens, (5) patients with initial presentation with poor histological subtype (mixed cellularity or lymphocyte depleted) extensive stage 3 or 4 and bulky disease (>5 cm) with B symptoms and (6) relapse after CR with previous ABMT. Marrow infiltration and performance status at transplantation were not necessarily exclusion criteria.

Patients with poor-prognosis primary disease who required urgent therapy for bulky obstructive disease could receive one course of conventional dose combination cytotoxic therapy prior to enrolment.

All patients gave informed consent, and the study was carried out in accordance with the principles of the Declaration of Helsinki.

Patient characteristics are shown in Tables I and II.

### Marrow harvest and conditioning regimen

Marrow (10 ml kg$^{-1}$ body weight) was harvested under general anaesthesia into heparinised M199 culture medium [marrow-culture medium, 1:1 (v/v)] and stored at 4°C. On recovery from anaesthesia the patient was returned to the ward, where melphalan 140 mg m$^{-2}$ (as a 1 h infusion) and etoposide 2.5 g m$^{-2}$ (infused at the rate of 500 mg l$^{-1}$ h$^{-1}$) were administered intravenously through a centrally placed Hickman catheter, with attention to fluid balance. Twenty-four hours after the completion of the chemotherapy the marrow was reinfused through the central line, after warming to 37°C. Two patients who achieved CR after HD-ABMT underwent a second procedure identical to the first (at 10

**Table I** High-dose melphalan plus VP16 with non-cryopreserved autologous bone marrow transplantation (ABMT) for Hodgkin's disease: patient and initial disease characteristics

| | *Number* | *Per cent* |
|---|---|---|
| Relapsed and refractory patients | | |
| Male | 29 | 66 |
| Female | 15 | 34 |
| Relapsed | 37 | 84 |
| Refractory | 7 | 16 |
| Disease stage at initial diagnosis | | |
| II | 8 | 18 |
| III | 18 | 41 |
| IV[a] | 18 | 41 |
| Systemic symptoms at initial diagnosis | | |
| A | 1 | 2 |
| B | 43 | 98 |
| Disease bulk at initial diagnosis | | |
| Bulky | 43 | 98 |
| Non-bulky | 1 | 2 |
| Initial treatment for Hodgkin's disease | | |
| MOPP | 15 | 34 |
| MOPP/ABVD | 12 | 27 |
| MOPP, salvage ABVD | 13 | 30 |
| ≥2 salvage regimens | 4 | 9 |
| Total nodal irradiation | 12 | 27 |
| Ethnic group | | |
| Black | 12 | 27 |
| White | 32 | 73 |
| Median time (months) to first relapse (±s.d. range) | 14 (2–35) | |
| Poor-prognosis HD receiving primary HDC+ABMT[b] | | |
| Female | 1 | 25 |
| Male | 3 | 75 |
| Stage III | 2 | 50 |
| Stage IV | 2 | 50 |
| B symptoms | 4 | 100 |
| Black | 4 | 100 |

[a]Seven patients had marrow involvement at initial presentation. [b]Of the four patients receiving primary treatment with BMT, two received one cycle of MOPP for debulking prior to BMT.

months and 12 months post initial HDC-ABMT) following relapse of the disease. The inclusion of these two patients resulted in 50 HDC-ABMT procedures in 48 patients.

In two patients, both of whom had marrow infiltration prior to transplantation, interferon-α (Intron A, Scherag SA) 20 million units was added to the bag at harvesting and marrow was separated and nucleated cells were cultured in M199 medium + 20% autologous serum in 180 $cm^2$ (Life cell Tissue Culture Flask; Fenwall Laboratories, Deerfield IL, USA) for 96 h at 37°C in 5% carbon dioxide. The mean number of nucleated cells infused was $2.77 \times 10^8$ $kg^{-1}$ with viability >85%. Further details are shown in Table III.

Patients were discharged 24 h after receiving the marrow reinfusion, and were followed as outpatients on a daily or alternate-day basis until either neutropenic pyrexia or severe mucositis occurred. All patients required readmission by day 8, and thereafter remained in hospital until recovery of the neutrophil count to ≤500. Although patients did not routinely receive growth factors, the option was available to use recombinant granulocyte–macrophage colony-stimulating factor (GM-CSF) as salvage therapy if engraftment did not occur by day 28.

### *Supportive care*

Patients received single donor irradiated platelets and filtered red cell transfusions as required, and all patients received prophylactic ciprofloxacin by mouth when neutropenia occurred. While in hospital, patients were nursed in single-bed private wards with reverse barrier nursing. Laminar airflow conditions were not used. Empirical antibiotic and antifungal therapy was utilised as indicated for neutropenic sepsis.

**Table II** High-dose melphalan plus VP16 with non-cryopreserved ABMT for Hodgkin's disease: characteristics at first BMT

| | *Number* | *Per cent* |
|---|---|---|
| Stage | | |
| III | 20 | 42 |
| IV | 28 | 58 |
| B symptoms | 48 | 100 |
| Bulky disease | 26 | 54 |
| Non-bulky disease | 22 | 41 |
| Histological subtype | | |
| NS | 23 | 48 |
| MC | 17 | 35 |
| LD | 8 | 17 |
| Site of involvement | | |
| Nodal/spleen | 21 | 44 |
| Liver | 9 | 19 |
| Lung | 14 | 29 |
| Marrow | 4 | 8 |
| CNS | 1 | 2 |
| Number of extranodal sites | | |
| 1–2 | 33 | 69 |
| ≥2 | 15 | 31 |
| Haemoglobin (g $dl^{-1}$) | | |
| ≥10 | 40 | 83 |
| <10 | 8 | 17 |
| Lymphocyte count ($\times 10^9$ $l^{-1}$) | | |
| ≥1.5 | 36 | 75 |
| <1.5 | 12 | 25 |
| Performance status (WHO) | | |
| 1 | 12 | 25 |
| 2 | 25 | 52 |
| 3 | 11 | 23 |
| Mean age ±s.d. (range) | 31 ± 4 (17–49) years | |

NS, nodular sclerosis; MC, mixed cellularity; LD, lymphocyte depleted.

**Table III** High-dose melphalan plus VP16 with non-cryopreserved ABMT for Hodgkin's disease: marrow collection data

| | *At marrow collection* | *At transplant* |
|---|---|---|
| | *Mean ± s.d.* | |
| Total nucleated cells $\times 10^8$ $kg^{-1}$ | 2.97 ± 1.3 | 2.77 ± 1.1 |
| $CD34^+$ cells $\times 10^6$ $kg^{-2}$ | 12.9 ± 5.9 | 12.3 ± 5.1 |
| Percentage viability | 93 ± 3 | 87 ± 2 |
| Mean storage time±s.d. (h)[a] | – | 36 ± 4 |

Forty-eight patients received 50 ABMTs. [a]Bone marrow of two patients with marrow involvement at the time of collection had *in vitro* bone marrow culture for 96 h with addition of IFN-α 20 mU.

### *Response criteria*

All patients were restaged 3 months after transplantation, and standard criteria to assess response were utilised.

### *Statistical analysis*

Response, toxic death, overall survival from date of transplantation and event-free survival were analysed using SAS statistical software. Factors which were examined for influence on response rate included sex, histological subtype, presence or absence of systemic symptoms, haemoglobin concentration, lymphocyte count, number of extranodal sites, presence or absence of marrow involvement, disease bulk, number of relapses, relapse status (relapsed versus refractory disease), the presence of liver involvement and performance status. Survival curves were estimated using the method of Kaplan and Meier, and were compared using the log-rank statistic.

**Table IV** High-dose melphalan plus VP16 with non-cryopreserved ABMT for Hodgkin's disease: haematological response

| | Number | Per cent |
|---|---|---|
| Haematological recovery[a] | 49 | 98 |
| Mean red cell transfusion requirement | 4 (±2) | |
| Mean platelet transfusion requirement | 4 (±2) | |
| | Median time to haematological recovery (days) | |
| Neutrophils $\geqslant 1.0 \times 10^9 l^{-1}$ | 22 | |
| Platelets $\geqslant 40 \times 10^9 l^{-1}$ | 25 | |

[a]Out of 50 bone marrow transplant procedures.

**Table V** High-dose melphalan plus VP16 with non-cryopreserved ABMT for Hodgkin's disease: toxicity

| | Number | Per cent |
|---|---|---|
| Early death | 1 | 2 |
| Intracerebral haemorrhage | 1 | 2 |
| Renal dysfunction | 2 | 4 |
| Nausea (grade 3–4) | 36 | 75 |
| Vomiting (grade 3–4) | 36 | 75 |
| Grade 3–4 mucositis | 48 | 100 |
| Neutropenic fever | 48 | 100 |

### Results

### *Response to treatment*

Forty-three of 48 patients (90%) achieved a complete response (CR), one (2%) patient achieved a partial response (PR), three patients showed no response to therapy (NR), and one patient was not eligible for evaluation (NE) owing to early death. Response rate according to a number of transplant procedures (50 procedures among 48 patients) was CR 43/50 (86%), PR 3/50 (6%), NR 3/50 (6%) and not evaluable 1 (2%). The two patients who relapsed after previous BMT both achieved only PR with the second transplant procedure. Of the factors examined for prognostic influence, only performance status ($\chi^2 = 14.61$, $P = 0.002$) and liver involvement ($\chi^2 = 11.58$, $P = 0.003$) were shown to significantly influence response to HDC-ABMT.

### *Toxicity*

Haematological recovery was documented in 49/50 (98%) transplant procedures (Table V). The one exception was a patient who died at day 10 post chemotherapy from multi-organ failure related to chemotherapy drug toxicity. This patient was classified as non-evaluable for haematological recovery because of early death.

One patient who was transplanted with extensive disease and a performance status of 3 died of early complications prior to haematological recovery. No other procedure-related mortality was experienced. Two patients had moderate reversible renal toxicity and one patient had a minor intracerebral haemorrhage, from which he made a full recovery (Table V).

All patients experienced severe grade 3–4 mucositis, which was usually the major cause of readmission to hospital. This necessitated the use of both intravenous analgesia and fluid for 3–4 days, and occasionally the use of hyperalimentation. All patients experienced grade 4 neutropenia and thrombocytopenia. The mean time to neutrophil count recovery ($\geqslant 1 \times 10^9$) was $23 \pm 7$ days (range 13–42 days) and the mean time to platelet recovery ($\geqslant 40 \times 10^9$) was $27 \pm 9$ days (range 15–60 days). All patients developed neutropenic fever requiring broad-spectrum intravenous antibiotics.

### *Survival*

The median survival has yet to be reached. Actuarial survival is shown in Figure 1. Factors analysed for influence on

**Figure 1** Actuarial survival of all patients with relapsed/refractory Hodgkin's disease treated with single high-dose melphalan (140 mg m$^{-2}$) and VP16 (2.5 g m$^{-2}$) plus non-cryopreserved autologous bone marrow transplantation.

**Figure 2** Influence of hepatic involvement on survival of patients with refractory/relapsed HD treated with high-dose melphalan + VP16 and ABMT. (●) patients without hepatic involvement; (○) patients with hepatic involvement ($P = 0.05$, log-rank).

survival included all those analysed for response to treatment. Univariate analysis indicated that refractory disease at transplantation ($\chi^2 = 9.12$, $P = 0.01$), hepatic involvement (Figure 2) ($\chi^2 = 3.76$, $P = 0.05$) and performance status ($\chi^2 = 3.72$, $P = 0.05$) were the only significant predictors of survival. The number of relapses prior to transplantation was not a significant factor predicting for either response or survival duration. The presence of marrow infiltration, either at time of transplantation or at initial presentation, also failed to influence survival. Thirty-nine patients are alive and 37 of these remain in unmaintained remission. Eight patients have relapsed and died of progressive disease, including two patients who received second ABMTs. Both of these patients achieved a second CR with survivals thereafter of 6 and 12 months.

### Discussion

Although the management of Hodgkin's lymphoma has achieved substantial success, with the majority of patients, including even those with extensive disease, attaining durable complete responses using conventional dose combination chemotherapy, there remains a significant proportion of patients who are either refractory to induction therapy or who relapse soon after achieving response. In addition, recent studies suggest that patients who have a poor prognosis with conventional dose chemotherapy (Fisher *et al.*, 1979) may be defined by prognostic criteria which include not only stage but also non-stage-related factors such as histological subtype, age, disease bulk, haemoglobin level and total lymphocyte count (Proctor *et al.*, 1992).

Non-cross-resistant multidrug chemotherapy regimens offer some hope of salvage in these patients, but the long-term disease-free remission and ultimate cure rates remain low. The most promising results have been obtained with the use of high-dose chemotherapy conditioning regimens with autologous bone marrow rescue (HDC-ABMT). Such poor-prognosis patients may well be candidates for early HDC-ABMT. Early reports, even in patients with refractory disease, suggested that complete response (CR) rates in excess of 50% could be achieved (Carella *et al.*, 1985). Although toxic death rates were fairly high in initial studies (3–80%) (Lu *et al.*, 1983; Goldstone *et al.*, 1985), this could probably be attributed to the fact that the majority of the reported patients had been extensively pretreated and were largely in resistant relapse. In addition, many patients had been previously exposed to thoracic radiotherapy and/or to other agents causing pulmonary toxicity. Such patients have a significantly increased risk of developing respiratory failure due to infection and pulmonary alveolar haemorrhage (Jules-Elysee *et al.*, 1992).

More recent studies such as that from Chopra *et al.* (1993) using a combination of BCNU, etoposide, cytosine arabinoside and melphalan (BEAM) have reported improved results with an approximately 10% prevalence of toxic death and a 50% actuarial 5-year relapse-free survival. Interestingly, these investigators reported a poorer survival not only in patients with refractory disease, but also those in first relapse, with patients in second and third relapse having a superior relapse-free survival.

HDC-ABMT appears to be relatively well tolerated, with good quality of life being attained, especially in responding patients (Chao *et al.*, 1992). Indeed, 96% of patients indicate that they would be willing to undergo the procedure a second time if necessary (Vose *et al.*, 1992).

To a large extent, HDC-ABMT procedures have utilised BCV- or BEAM-like regimens given over a number of days and necessitating cryopreservation of the autologous marrow (Reece *et al.*, 1991). Optimum treatment regimens and schedules remain to be defined however. Treatment programmes utilising agents such as melphalan and etoposide, which have short half-lives, can be administered in less than 24 h with the potential advantage of not requiring cryopreservation of marrow (Carella *et al.*, 1985; Taylor *et al.*, 1993), thus making the procedure less costly and technically less demanding.

There is also controversy as to whether HDC-ABMT should be used as the initial treatment approach for relapsed and refractory disease, or whether cytoreductive therapy should be attempted prior to ABMT. An advantage of initial cytoreduction is that chemosensitivity can be assessed prior to transplantation. Numerous investigators have found sensitive relapse to be predictive of remission rate and longer disease-free survival following HDC-ABMT (Crump *et al.*, 1993*b*). However, if the approach is to be HDC-ABMT whatever the outcome of cytoreductive therapy, an obvious disadvantage of attempting retreatment prior to HDC-ABMT would be a more heavily pretreated patient undergoing ABMT, with a resultant increase in morbidity and possibly mortality.

The regimen that we have utilised has a number of advantages. No specialised marrow handling equipment, cryopreservation or storage techniques are required, as the marrow is merely stored at 4°C and reinfused 36–48 h after harvesting. The chemotherapy regimen is uncomplicated and can be administered in a single day, thus allowing the discharge of the patient for 4–7 days prior to the onset of mucositis and neutropenia, with resultant cost savings.

Although morbidity from severe mucositis was high, occurring in all patients, it generally necessitated an inpatient stay of between 7 and 14 days only, and was easily managed. Only one early death occurred, and other morbidity was generally self-limiting.

Whether the use of bone marrow culture with interferon alpha added anything to the treatment of patients with marrow involvement at the time of BMT cannot be determined from the current study. The presence of marrow involvement is obviously a worrying feature with the possibility of returning a contaminated graft. Other possible methods of dealing with this problem may be some positive selection procedure, selecting for multipotent (stem cell) bone marrow precursors only. However, the utility of and even theoretical background for such procedures must wait for a clearer definition of the characteristics of the malignant cell in HD.

One further aspect that deserves comment is the question of the use of bone marrow as against peripheral blood precursor cells (PBPCs) for effecting haematological rescue. A number of studies have shown rapid haematological recovery following HDC with growth factor-mobilised PBPCs in a variety of haematological and non-haematological malignancies (To *et al.*, 1992; Crump *et al.*, 1993*a*; Pettengel *et al.*, 1993*a*, *b*). The studies of Pettengel *et al.* suggest more rapid haematological engraftment with PBPCs, but whether BMT or PBPCs is the most cost-effective rescue procedure in patients with HD requires further study.

Despite these provisos it should, however, be pointed out that a 90% complete response rate was achieved and median survival and median time to treatment failure have yet to be reached. While a poor performance status and hepatic involvement appeared to be adverse prognostic factors for response, a number of patients with these features did achieve CR and substantial disease-free survival. Relapse status at time of transplant, whether first, second or third relapse, did not appear to affect either response rates or survival.

The regimen and therapeutic approach described appears to offer response rates and survival at least equivalent to those reported in the literature, and is in addition brief and uncomplicated, requiring little specialised equipment and a shortened inpatient hospital stay. There appears to be no advantage to initial cytoreductive therapy in patients with Hodgkin's disease who require ABMT and high-dose chemotherapy, except in patients who require urgent therapy for obstructive complications.

### References

BEZWODA, W.R., MACPHAIL, A.P., DANSEY, R., SEYMOUR, L., SITAS, F., COHN, R. & POOLE, J. Hodgkin's Disease and its treatment in sub-Saharan Africa (in press).

CARELLA, A.M., SANTINI, G. & SANTORO, A. (1985). Massive chemotherapy and non-frozen autologous bone marrow transplantation in 123 cases of refractory Hodgkin's disease. *Eur. J. Cancer Clin. Oncol.*, **21**, 607–613.

CARELLA, A.M., CARLIER, P., CONGIN, A., GAOZZA, E., OCCHINI, D., MELONI, G., ANSELMO, A.P., MANDELLI, F., MAZZA, P., TURA, S. & 11 others (1991*a*). Nine years experience with ABMT in 128 patients with Hodgkin's. An Italian Study Group Report. In *Fifth International Symposium: Autologous BMT*, Dicke, K.A. & Armitage, J.O. (eds) pp. 509–518. Omaha: The University of Nebraska Medical Centre.

CARELLA, A.M., CARLIER, P., CONGUI, A., OCCHINI, D., NATI, S., PIER-LIUGI, D., GIORDAMO, D., BACIGALUPO, A. & DAMASIO, E. (1991b). Autologous BMT as adjuvant treatment for high-risk Hodgkin's disease in first remission after MOPP/ABVD protocol. *Bone Marrow Transplant.*, **8**, 99–103.

CHAO, N.J., TIERNEY, D.K., BLOOM, J.R., LONG, G.D., BARR, T.A., STALLBAUM, B.A., WONG, R.M., NEGRIN, R.S., HORNING, S.J. & BLUME, K.G. (1992). Dynamic assessment of quality of life after autologous bone marrow transplantation. *Blood*, **80**, 825–830.

CHOPRA, R., MCMILLAN, A.K., LINCH, D.C., YUKLEA, S., TAGHIPOUR, G., PEARCE, R., PATTERSON, G. & GOLDSTONE, A.H. (1993). The place of high dose BEAM therapy and autologous bone marrow transplantation in poor risk Hodgkin's disease. A single centre 8 year study of 155 patients. *Blood*, **82**, 1137–1145.

CRUMP, M., ROSS, M., VREDENBURGH, J., SHPALL, E., JONES, R., MEISENBURG, B. & PETERS, W.P. (1993a). Phase II evaluation of high-dose thiotepa, cisplatin and cyclophosphamide with autologous stem cell rescue for patients with refractory solid tumours (abstract). *Proc. Am. Soc. Clin. Oncol.*, **12**, 13.

CRUMP, M., SMITH, A., BRADWEIN, J., COUTURE, F., SHERRET, H., SUTTON, D., SCOTT, J., MCCRAE, J., MURRAY, C., PANTALONY, D., SUTCLIFFE, S. & KEATING, A. (1993b). High dose etoposide and melphalan and autologous bone marrow transplantation for patients with advanced Hodgkin's disease: importance of disease status at transplantation. *J. Clin. Oncol.*, **11**, 704–711.

FISHER, R.I., DE VITA, V.T., HUBBARD, S.P., SIMON, R. & YOUNG, R.C. (1979). Prolonged disease free survival in Hodgkin's disease with MOPP reinduction after first relapse. *Ann. Int. Med.*, **90**, 761–763.

GOLDSTONE, A.H., ANDERSON, C.C. & SOUHAMI, R. (1985). Very high dose chemotherapy in adult patients with resistant relapsed lymphoma. *Int. J. Cell Cloning*, **3**, 225–228.

GRIBBEN, J.G., GOLDSTONE, A.H. & LINCH, D.C. (1989). Preliminary results of ABMT in the management of resistant Hodgkin's disease: experience of the Bloomburg Transplant Group at University College, London. *Recent Results Cancer Res.*, **117**, 242–247.

JULES-ELYSEE, K., STOVER, D.E., YAHALOM, J., WHITE, D.A. & GULATI, S.C. (1992). Pulmonary complications in lymphoma patients treated with high dose therapy and autologous bone marrow transplantation. *Am. Rev. Respir. Dis.*, **146**, 485–491.

LU, C., BRAINE, H.G. & KAIZER, H. (1983). Preliminary results of high dose busulphan and cyclophosphamide with syngeneic or autologous bone marrow rescue. *Cancer Treat. Rep.*, **68**, 711–717.

PETTENGEL, R., TESTA, N.G., SWINDELL, R., CROWTHER, D. & DEXTER, T.M. (1993a). Transplantation potential of hemopoietic cells released into the circulation during routine chemotherapy for non-Hodgkin's lymphoma. *Blood*, **82**, 2239–2248.

PETTENGEL, R., MOGENSTERN, G.R., WOLL, P.J., CHANG, J., ROWLANDS, M., YOUNG, R., RADFORD, J.A., SCARFFE, J.H., TESTA, N.G. & CROWTHER, D. (1993b). Peripheral blood progenitor cell transplantation in lymphoma and leukemia using a single apheresis. *Blood*, **82**, 3770–3777.

PROCTOR, S.J., TAYLOR, P., MACKIE, P., DONNAN, P., BOYS, R., LENNARD, A. & PRESCOTT, R.J. (1992). A numerical prognostic index for clinical use in identification of poor risk patients with Hodgkin's disease at diagnosis. *Leukaemia Lymphoma*, **7**, 17–20.

REECE, D.E., BARNETT, M.J., CONNORS, J.M., FAIREY, R.N., GREER, J.P., HERZIG, G.P., HERZIG, R.H., KLINGEMANN, H.-G., O'REILLY, S.E., SHEPHERD, J.D., SPINELLI, J.J., VOSS, N.J., WOLFF, S.N. & PHILLIPPS, G.L. (1991). Intensive chemotherapy with cyclophosphamide, carmustine and etoposide followed by autologous bone marrow transplantation for relapse Hodgkin's disease. *J. Clin. Oncol.*, **9**, 1872–1878.

SULLIVAN, K.M., APPELBAUM, F.R., HORNING, S.J., ROSENBERG, S. & THOMAS, E.D. (1986). Selection of patients with Hodgkin's disease and non-Hodgkin's lymphoma for bone marrow transplantation. *Int. J. Cell Cloning*, **4** (Suppl. 1), 94–106.

STRAUSS, D.J., GAYNER, J.J. & MYERS, J. (1990). Prognostic factors among 185 adults with newly diagnosed advanced Hodgkin's disease treated with alternating potentially non-cross resistant chemotherapy and intermediate dose radiation therapy. *J. Clin. Oncol.*, **7**, 1173–1181.

TAYLOR, P.R.A., JACKSON, G.H., LENNARD, A.L., LUCRAFT, H. & PROCTOR, S.J. (1993). Autologous transplantation in poor risk Hodgkin's disease using high dose melphalan/etoposide conditioning with non-cryopreserved marrow rescue. *Br. J. Cancer*, **67**, 383–387.

TO, L.B., ROBERTS, M.M., HAYLOCK, D.N., DYSON, P.G., BRANFORD, A.L., THORP, D., HO, J.Q.K., DART, G.W., HORVATH, N., DAVY, M.L.J., OLWENY, C.L.M., ABDI, E. & JUTTNER, C.A. (1992). Comparison of hematological recovery times and supportive care requirements of autologous recovery phase peripheral blood stem cell transplants, autologous bone marrow transplant and allogeneic bone marrow transplants. *Bone Marrow Transplant.*, **9**, 277–283.

VOSE, J.M., KENNEDY, B.C, PIERMAN, P.J., KESSINGER, A. & ARMITAGE, J.O. (1992). Long term sequelae of autologous bone marrow or peripheral stem cell transplantation for lymphoid malignancies. *Cancer*, **69**, 784–789.